# Comparative analysis of the labelling of nanotechnologies across four stakeholder groups

**DOI:** 10.1007/s11051-015-3129-8

**Published:** 2015-07-29

**Authors:** Adam Capon, James Gillespie, Margaret Rolfe, Wayne Smith

**Affiliations:** Menzies Centre for Health Policy, School of Public Health, University of Sydney, The University of Sydney, NSW 2006 Australia; Environmental Health Branch, Health Protection NSW, Sydney, Australia; University Centre for Rural Health, School of Public Health, University of Sydney, Sydney, Australia

**Keywords:** Nanotechnologies, Labelling, Stakeholder, Risk perception

## Abstract

Societies are constantly challenged to develop policies around the introduction of new technologies, which by their very nature contain great uncertainty. This uncertainty gives prominence to varying viewpoints which are value laden and have the ability to drastically shift policy. The issue of nanotechnologies is a prime example. The labelling of products that contain new technologies has been one policy tool governments have used to address concerns around uncertainty. Our study develops evidence regarding opinions on the labelling of products made by nanotechnologies. We undertook a computer-assisted telephone (CATI) survey of the Australian public and those involved in nanotechnologies from the academic, business and government sectors using a standardised questionnaire. Analysis was undertaken using descriptive and logistic regression techniques. We explored reluctance to purchase as a result of labelling products which contained manufactured nanomaterials both generally and across five broad products (food, cosmetics/sunscreens, medicines, pesticides, tennis racquets/computers) which represent the broad categories of products regulated by differing government agencies in Australia. We examined the relationship between reluctance to purchase and risk perception, trust, and familiarity. We found irrespective of stakeholder, most supported the labelling of products which contained manufactured nanomaterials. Perception of risk was the main driver of reluctance to purchase, while trust and familiarity were likely to have an indirect effect through risk perception. Food is likely to be the greatest product impacted by labelling. Risk perception surrounding nanotechnologies and label ‘framing’ on the product are key issues to be addressed in the implementation of a labelling scheme.

## Introduction

Societies are constantly faced with a range of risks and are challenged to develop the appropriate policy responses. Part of the challenge lies with the fact that the management of risks are value laden, and their assessment is likely to be multidisciplinary in nature (Aven and Zio 2014; Hansson and Aven 2014). The differing values and interpretations of ‘risk’ both within disciplines (Andretta 2014; Aven and Zio 2014) and more broadly society as a whole can lead to contrasting positions. The balancing of these positions is how policy is formed; however, a sudden shift in the power of one position can lead to significant regulatory and policy reform (Hood et al. 2001) with potentially undesirable consequences. Therefore, understanding societal perceptions of risk and risk values is important to ensure a balanced policy approach.

Nanotechnologies is described as a collective term for a range of technologies, techniques and processes that involve the manipulation of matter at the nanoscale—the sizes range from approximately 1 nanometre to 100 nanometres (Australian Office of Nanotechnology 2007), and is a new technology being introduced into society. Past experiences of introducing new technologies into society has highlighted the importance of how the technology is introduced. The introduction when poorly done, as was the case for genetically modified organisms (GMO) into Europe, has led to societal rejection of the technology (Devos et al. 2006; Vogel 2012). Concerns have been raised that the introduction of nanotechnologies into society could follow the same path as that of GMO in Europe (Duncan 2011; Siegrist 2010). To avoid this fate, many have prompted calls for greater public engagement and early consideration of regulatory tools such as labelling for nanotechnologies (Bostrom and Löfstedt 2010; D’Silva and Bowman 2010; Siegrist 2010).

Labelling is a tool that easily conveys information to consumers so they can make an informed choice with regard to a product (D’Silva and Bowman 2010). Support for the labelling of nanotechnology-based products centres on societal rights that will ultimately bring about consumer acceptance of nanotechnologies. Specifically, it is argued that every citizen has a right to know what they are purchasing. This right then provides them with freedom of choice to purchase or not to purchase the product. This increased transparency will lead to greater trust between consumer and producer, which will ultimately lead to greater acceptance and less resentment of nanotechnology-based products. It is also argued that labelling allows consumers to become aware, and through this awareness, informed about nanotechnology-based products. Finally, labelling provides the ability for nanotechnology-based products to be tracked through society allowing researchers and governments the increased ability to determine and control for any adverse effects they may have on society (Adler 2010; Brown and Kuzma 2013; D’Silva and Bowman 2010; Gruère 2011; Morris et al. 2011).

Mandatory consumer product labelling has always been a point of contention between regulatory agencies, businesses and the public (D’Silva and Bowman 2010). Arguments against the labelling of nanotechnology-based products centre on misinterpretation of label meaning and the complications of implementing a labelling scheme. Specifically, arguments include negative perceptions (creating stigma, fear, reduced public acceptance and disinvestment), additional costs to implement labelling, potential trade barriers between the country that requires labelling and those who do not, shifting the responsibility of determining the acceptability of the risk to the consumer because now they have a choice, and public apathy. Complications of implementation of a scheme include a clear definition of nanoproducts, having creditable and accurate detection tools to enforce the system, having clear guidance regarding thresholds for accidental (and natural) nanomaterial contamination, and clear guidance around a product that may not contain nanoproducts, but which has been manufactured by nanoproducts (Adler 2010; Brown and Kuzma 2013; D’Silva and Bowman 2010; Gruère 2011; Stamm 2011). Despite these challenges, Bowman et al. (Bowman et al. 2010) and Stamm (Stamm 2011) provide some insight into how this regulation is undertaken in the EU, with the EU (Aschberger et al. 2014), New Zealand (New Zealand Government 2012) and Australia (Australian Government 2011) requiring compulsory labelling of certain nanoproducts.

The few peer-reviewed studies of public perceptions of labelling products containing manufactured nanomaterials have shown the public is generally supportive of their labelling, regardless of origin of population studied (Brown and Kuzma 2013; IPSOS Social Research Institute 2012; Throne-Holst and Rip 2011). Australian studies have found that the public is concerned about the lack of potential regulations/testing for labelling (Market Attitude Research Services 2011) and supportive of government funding to rectify this (IPSOS Social Research Institute 2012). In a Norwegian study, participants highlighted concern regarding a regulatory framework that did not fully cover nanoproducts (Throne-Holst and Strandbakken 2009). This study drew out three key concerns: transparency (about the motives and criteria for labelling), accountability (those who set up the programme need to be accountable for it) and responsibility (someone needs to be responsible for it) (Throne-Holst and Rip 2011; Throne-Holst and Strandbakken 2009). A study in the United States showed the public have concerns regarding regulation, although they believed they would have little influence in the development of such regulation (Brown and Kuzma 2013). This study drew out three themes: “Labelling Preference”—most were in support for it and suggested it be on the front side of packaging; “Label Use Moderators”—the label will require explanation for the public to use it correctly; and “Information Sources”—two dichotomous themes emerged, institutionally based and personally based information sources, i.e. some saw it was the responsibility of government and industry to provide the education, while others saw it as a personal responsibility. The study further found that participants rely heavily on past experiences of product labelling in order to inform opinions on nanolabelling and concluded that consumer acceptance of the product relates to its ultimate success (Brown and Kuzma 2013). A Swiss study examined the influence the labelling of sunscreen bottles had on the perceptions of risk and found that the label significantly increased risk and reduced benefit perception (Siegrist and Keller 2011). However, in this study, it could be argued that the ‘framing’ of the labelling could have biased the perceptions, be that its size, shape and position on the bottle, was alarming in itself. In contrast, *mandatory labelling of consumer cosmetics within Europe has resulted in very little societal response* (Frewer et al. 2014).

In this paper, we develop evidence currently lacking in the international published literature. That is, different stakeholder perceptions of labelling products made from nanotechnologies along with the factors associated with these perceptions. We explore the relationships between Australian public, academic, government and business risk and labelling perceptions around nanotechnologies and investigate the potential advantages, barriers and impacts of labelling products containing manufactured nanomaterials. We undertake this on five generic product-based items (food, cosmetics/sunscreens, medicine, pesticides, and tennis racquet/computers) which represent the broad categories of products regulated by differing government agencies in Australia (Capon et al. 2013). We have a number of hypotheses. The public perception of risk from nanoparticles is much higher than that expressed by other stakeholders (Capon et al. 2015a; Siegrist et al. 2007). Also a person’s risk perception can influence their purchasing habits with evidence that a negative risk perception is associated with negative purchasing habits (Verbeke et al. 2007; Yeung and Morris 2001). Accordingly, our first two hypotheses are that the public will be significantly less likely to buy a product if it has a label on it stating it contains nanomaterials than any other stakeholder. Further, those in the public who perceive nanoparticles as a risk in a particular generic product will be significantly less likely to buy that product if it has a label stating it contained manufactured nanoparticles. When faced with unresolved risk, consumers will draw on a range of strategies including endorsement from trusted sources (Yeung and Morris 2001). Therefore, our third hypothesis is that those who have less trust in the health department, scientists, politicians and journalists to keep them safe from any possible adverse health effects of nanomaterials will be significantly less likely to buy a product if it has a label on it stating it contains nanomaterials. Low familiarity of nanotechnology has been associated with the increased perception of risk of manufactured nanomaterials (Capon et al. 2015a). Subsequently, our fourth hypothesis is that those who report lower familiarity with nanotechnology will be significantly less likely to buy a product if it has a label on it stating it contains nanomaterials.

## Methods

### Participation details

In March 2013, we undertook a nationally representative cross-sectional household survey (*n* = 1355) of adult individuals using computer assisted telephone interviewing (CATI) landline (response rate = 34 %) and mobile phone technologies (response rate = 19 %). Sampling was based on random selection from a stratified area probability sample of private dwellings and mobile phone users. All participants were recruited through random digit dialling sampling with landline respondents selected through the ‘last birthday technique’. Sample weights accounted for the probability of selection, calibrated by age and gender (but not for jurisdictional strata) to the June 2012 Australian Bureau of Statistics (ABS) Estimated Resident Population.

From May–July 2013, we undertook a similar survey of academic, business and government stakeholders using CATI landline technology. Academic and business contacts were identified if they belong to the Australian Nanotechnology Network members list or are named in the 2011 Nanotechnology—Australian Capability Report 4th edition. Government contacts were identified through snowballing technique instigated by one of the authors who is actively involved in the area. A total of 1732 academic, 69 business and 45 government contacts were identified. All identified business (response rate = 36 %, *n* = 21) and government contacts (response rate = 48 %, *n* = 19) were approached, while a simple random sample of academic contacts (response rate = 33 %, *n* = 301) through random sorting and selection was undertaken.

### Survey design/measures

The survey was developed, from previous studies (IPSOS Social Research Institute 2012; McAllister 2011; Retzbach et al. 2011; TNS Opinion and Social 2010) and had cognitive testing on 10 random individuals of varying ages and gender, following Australian Bureau of Statistics guidelines (Population Survey Development 2001). Final survey measures were chosen based on cognitive understanding and easing of respondent fatigue and subject to expert review. Common survey questions were repeated verbatim across all surveys and in the same order to ensure comparability of answers, and order of questions within a topic were randomised to avoid ordering effects. Participants were given an introduction to nanotechnology and manufactured nanomaterials, presented in a neutral fashion to minimise any bias. They were told “Nanotechnology is science at a very small scale and refers to a new array of devices and materials whose key parts are 10,000 times smaller than the width of a human hair. Working at this scale allows science researchers to create new materials and products”, “Manufactured nanomaterials are the minute particles produced from nanotechnology. They are found in over 1000 products on the world market today including some food containers, cosmetics and sunscreens, clothing, sporting goods and computers.” The primary outcome variables of interest for measuring reluctance to purchase due to labelling was based on the response to the questions “If a (food product), (sunscreen), (non-prescription (off the shelf) medicine), (pesticide), (consumer good such as a tennis racquet or computer) had a label on it stating it contains nanomaterials would you be less likely to buy it?”. Answers were binary: yes/no.

Other variables of interest included basic demographics (age, gender), corresponding ‘nano risk’, general opinion on labelling and ‘nano trust’ (of the health department, scientists, journalists and politicians). The public survey included ‘nano familiarity’, while the academic, business, government survey included open-ended questions on challenges and advantages of labelling products containing manufactured nanomaterials.

Corresponding ‘nano risk’ was determined in response to the questions “Overall, in my opinion, manufactured nanomaterials are a risk if they are put in the food I eat” or “Overall in my opinion, putting manufactured nanomaterials into products such as (cosmetics and sunscreens) (medicines) (pesticides) (tennis racquets and computers) is a risk.” and was categorised into agree versus disagree. Respondents were asked to consider both risks AND benefits when answering these questions.

General opinion on labelling was determined in response to the following question “Some people say that we should put labels on products to let them know that they contain manufactured nanomaterials. What do you think? Do you think they should be labelled or they should not be labelled?”. Answers were binary: yes/no.

‘Nano trust’ was determined in response to the question “In general how much do you trust X to keep you safe from any possible health effects of nanomaterials? Would you say you have no trust at all, a little trust, moderate trust, a lot of trust or absolute trust?”. These answers were then collapsed into a three-point ‘low’ (no—a little), ‘moderate’ (moderate) and ‘high’ (a lot—absolute) response.

‘Nano familiarity’ was based on a composite of three questions. “Before today, had you heard of the term ‘Nanotechnology’” (Yes/No), if yes “Have you ever talked about nanotechnology with anyone before today?”, “Have you ever searched for information about nanotechnology?” (Yes, frequently; Yes, occasionally; Yes, only once or twice; No, never). No familiarity (*n* = 398) was based on a “no” answer to the first question, moderate familiarity (*n* = 422) on a “yes” answer to all questions, while some familiarity (*n* = 528) was the composite of the remaining answers.

Challenges and advantages with labelling were determined from two open-ended questions. “Do you see any challenges or problems with labelling products which contain manufactured nanomaterials? (‘Yes’/‘No’). If ‘Yes’ “Can you please tell me what you see are the challenges or problems with labelling products which contain manufactured nanomaterials?”. “Do you see any advantages or positive outcomes with labelling products which contain manufactured nanomaterials?” (‘Yes’/‘No’). If ‘Yes’ “Can you please tell me what you see are the advantages or positive outcomes with labelling products which contain manufactured nanomaterials?”.

The final survey instrument received ethics approval from the University of Sydney Ethics Committee. (2012/1841).

### Statistics

Data were analysed using SAS Enterprise Guide 6.1 with the Proc Survey function. The Proc Survey function allows analysis to be corrected for weighting and stratified sample design to provide prevalence estimates. Statistical significance between all four stakeholders was determined at a *P* value of ≤0.05 and is reported as such in this paper. For the analysis of public data, only a secondary *P* value of ≤0.01 was also considered to account for multiple comparisons. The sample size for business and government respondents were not large enough to consider the *P* value of ≤0.01.

Prevalence estimates of reluctance to purchase due to labelling and risk perceptions were analysed using the descriptive statistics of proportions.

Thematic analysis was undertaken by one coder on the open-ended questions regarding the challenges and advantages of labelling products containing manufactured nanomaterials to systematically identify the scope of issues. Themes were identified on a subset of 90 answers and examined across all answers. Where a subsequent theme was identified outside the subset of 90 answers, this theme was then examined across the whole dataset.

Two-by-two table analysis was undertaken to determine associations between reluctance to purchase due to labelling and ‘nano risk’ for the public survey data.

To examine the relationship between stakeholder groups and reluctance to purchase, an unadjusted logistic model was used for comparing reluctance to purchase each product type (5) across the four stakeholder groups. In addition, the same logistic models were adjusted for ‘nanorisk’. The logistic models contained an event category equal to ‘yes’, for comparing the proportions that were less likely to buy (reluctant to purchase) between groups. The public were used as the reference group, where a value less than 1 signalled a lower proportion that were less likely to buy than the public and a value greater than 1 a higher proportion that were less likely to buy than the public.

To examine the relationship between reluctance to purchase by the public and trust the public have in all four trust actors an unadjusted logistic model was used for comparing reluctance to purchase each product type (5) and trust in each actor (4). In addition, the same logistic models were adjusted for ‘nanorisk’, age and gender. Finally, examination of the relationship between reluctance to purchase by the public and familiarity the public have with nanotechnology was undertaken using an unadjusted logistic model to compare reluctance to purchase each product type (5) between the three levels of familiarity. These models were then adjusted for ‘nanorisk’, age and gender.

All variables were retained in all adjusted logistic regression models as these variables were central to the purpose of the study (Agresti 2014). The Wald test was used to determine statistical significance, and significant effects are also indicated with odds ratio confidence intervals that do not cross the value of 1.

## Results

### Overview

In this study, we found that a substantial majority of public (95 %, CI 93–96 %), academic (83 %, CI 79–88 %), government (71 %, CI 46–95 %) and business (68 %, CI 45–91 %) respondents believed we should put labels on products to let people know they contain manufactured nanomaterials. For the public, the product most likely to be affected by labelling was food (73 % less likely to buy) followed by off-the-shelf medicines (57 %), cosmetics/sunscreens (55 %), pesticides (50 %) and computers/tennis racquets (29 %). This pattern was broadly consistent for all other stakeholders (Table 1).

**Table 1:** Prevalence, estimated for each stakeholder, of being less likely to buy the product if that product had nano labelling

Product	Public			Academic			Government			Business		
	Less likely %	95 % LCI	95 % UCI	Less likely %	95 % LCI	95 % UCI	Less likely %	95 % LCI	95 % UCI	Less likely %	95 % LCI	95 % UCI
Food	72.8	69.7	75.9	55.4	49.3	61.5	31.6	8.6	54.6	26.3	4.5	48.1
Off the shelf medicines	57.0	53.6	60.4	35.4	29.9	41.0	25.0	1.2	48.8	10.5	0.0	25.7
Sunscreen	54.7	51.2	58.1	26.8	21.7	31.9	22.2	0.9	43.5	4.8	0.0	14.7
Pesticides	49.3	45.8	52.7	29.8	24.5	35.2	22.2	0.9	43.5	23.8	3.9	43.7
Tennis racquet/computer	28.9	25.8	32.0	6.5	3.6	9.3	5.3	0.0	16.3	4.8	0.0	14.7

Of the 341 academic, government and business respondents, 233 acknowledged challenges with the labelling of products containing manufactured nanomaterials. Of the 233, by far the greatest mentioned challenges were the creation of a negative perception for nanomaterials (43 %), followed by a lack of knowledge/understanding of nanomaterials to interpret what the label meant (30 %), and then lack of information contained on the label to inform the public of specific risks (27 %). Other challenges raised included the ambiguity of what nanotechnology is, including how to define and identify nanomaterials and the changing nature of nanomaterials through their lifecycle. A lack of regulation and regulatory framework to oversee the labelling process and the inability to deliver what the public expect from a labelling system were also raised, as were problems with educating the public on what the labels meant, consumers ignoring the label, the cost of implementing a labelling system and industry resistance.

Of the 341 academic, government and business respondents, 279 acknowledged advantages with the labelling of products containing manufactured nanomaterials. Of these 279, the two most mentioned advantages included consumer awareness/education of a product that contained nanomaterials (48 %) and the consumer’s right to know, to have informed choice and to have input into how the technology should be released into society (35 %). Other advantages included increased transparency leading to social trust and acceptance, and the ability to trace products through society for safety purposes. Finally, some believed labelling could give products a competitive marketing advantage, showing they were advanced and cutting edge.

### Relationship between stakeholder regarding reluctance to purchase

Unadjusted logistic analysis found the public were significantly less likely to buy food, sunscreens, off-the-shelf medicines and pesticides than academic, business and government stakeholders if those products had a label on them stating they contained nanomaterials, and significantly less likely to buy tennis racquets/computers than academic and business stakeholders if they had a label on them stating they contained nanomaterials. However, after adjusting for risk perception, only significant differences remained between public and academic opinions for food, sunscreen, pesticides and tennis racquets/computers (Table 2).

**Table 2:** Logistic analysis of stakeholder perceptions with respect to being less likely to buy because of labelling (yes versus no, where yes is the event category)

Labelling of product	Stakeholder	Crude odd ratio	95 % LCI	95 % UCI	Adjusted odds ratio	95 % LCI	95 % UCI
Food	Public	1 (Ref)			1 (Ref)		
	Academic	0.3	0.3	0.4	0.6	0.4	0.9
	Government	0.2	0.1	0.5	0.5	0.3	1.1
	Business	0.1	0.1	0.4	0.7	0.2	2.6
Sunscreen	Public	1 (Ref)			1 (Ref)		
	Academic	0.3	0.2	0.4	0.5	0.3	0.7
	Government	0.2	0.1	0.8	0.4	0.1	1.2
	Business	0.04	0.01	0.3	0.2	0.02	1.1
Off the shelf medicines	Public	1 (Ref)			1 (Ref)		
	Academic	0.4	0.3	0.6	0.8	0.5	1.1
	Government	0.3	0.1	0.8	0.7	0.2	2.2
	Business	0.09	0.02	0.4	0.3	0.1	1.2
Pesticides	Public	1 (Ref)			1 (Ref)		
	Academic	0.4	0.3	0.6	0.6	0.4	1.0
	Government	0.3	0.1	0.9	0.6	0.2	1.7
	Business	0.3	0.1	0.9	0.7	0.4	1.5
Tennis racquet or computer	Public	1 (Ref)			1 (Ref)		
	Academic	0.2	0.1	0.3	0.4	0.2	0.8
	Government	0.1	0.02	1.1	0.3	0.04	1.8
	Business	0.1	0.02	1.0	0.5	0.1	4.4

### Relationship between the public’s perception of risk and reluctance to purchase

For all the five products, we found the prevalence of the population that thought manufactured nanomaterials in a product were a risk was significantly greater than the prevalence of those who were reluctant to purchase because of labelling. This is shown in Table 3 which presents estimated prevalence of the public opinion regarding risk of manufactured nanomaterials in a particular product with the estimated prevalence of the public who reported they would be less likely to buy that product if it had a label on it stating it contains nanomaterials.

**Table 3:** Prevalence of the public opinion regarding risk of nanomaterials in a product/would be less likely to buy that product if it had a label on it

Product	Risk (agree)			Less likely to buy (yes)		
	%	99 % LCI	99 % UCI	%	99 % LCI	99 % UCI
Food	84.8	81.5	88.1	72.8	68.7	76.8
Cosmetics/sunscreen	72.2	68.0	76.4	54.7	50.2	59.2
Medicines	70.8	66.5	75.0	57.0	52.5	61.5
Pesticides	63.8	59.2	68.4	49.3	44.7	53.8
Tennis racquet/computer	39.6	35.1	44.1	28.9	24.9	33.0

A positive association was found between a perception of risk of manufactured nanomaterials in a product and the reporting of being less likely to buy that product if it had a label stating it contained manufactured nanomaterials. This association held for all the five products (Table 4). Table 4 shows the section of the public that reported a belief that manufactured nanomaterials in food were a risk were 24 times less likely to buy a food product if it was labelled as containing nanomaterials. If they thought manufactured nanomaterials in cosmetics/sunscreens were a risk, they were 21 times less likely to buy a sunscreen if it had a label on it stating it contained nanomaterials. Similarly, those who thought manufactured nanomaterials in medicines were a risk were 9 times less likely to buy an off-the-shelf medicine labelled as containing nanomaterials. In the case of manufactured nanomaterials in pesticides, those who perceived risk were 15 times less likely to buy a pesticide labelled as containing nanomaterials. Finally, the smaller group who reported viewing manufactured nanomaterials in tennis racquets/computers as a risk, were 20 times less likely to buy a product if it had a label on it stating it contained nanomaterials.

**Table 4:** Two-by-two tables of perceived risk of nanomaterials in a product and effect of labelling

Risk (agree/disagree)	Less likely to buy (yes)	%	OR	99 % LCI	99 % UCI
Product					
Food (agree)	Food	84.2	24.2	12.4	47.1
Food (disagree)	Food	18.1			
Sunscreen (agree)	Sunscreen	73.4	21.1	11.4	39.2
Sunscreen (disagree)	Sunscreen	11.5			
Medicines (agree)	Medicines	73.4	9.2	5.7	14.8
Medicines (disagree)	Medicines	23.1			
Pesticides (agree)	Pesticides	71.3	14.9	8.6	25.9
Pesticides (disagree)	Pesticides	14.3			
Tennis racquet/computer (agree)	Tennis Racquet/computer	62.1	19.5	11.1	34.0
Tennis racquet/computer (disagree)	Tennis Racquet/computer	7.8			

### Relationship between the public’s trust in various actors and reluctance to purchase

Table 5 shows the results of unadjusted logistic analyses between the public’s trust in various actors and reluctance to purchase induced by labelling. A value less than 1 indicates a greater likelihood to purchase. It shows that lower trust in scientists was significantly associated with being less likely to buy food, sunscreen, off-the-shelf medicines, pesticides and tennis racquets/computers if it had a label on it stating it contained nanomaterials. Lower trust in the health department was significantly associated with being less likely to buy sunscreen, off-the-shelf medicines and tennis racquets/computers if it had a label on it stating it contained nanomaterials. Lower trust in politicians was significantly associated with being less likely to buy sunscreen if it had a label on it stating it contained nanomaterials. However, after adjusting for risk perception, age and gender, trust the public had in any actor (health department, scientist, journalist or politician) was not associated with being less likely to buy any product, with risk perception being the main influence of whether people were less likely to buy or not (*P* < 0.01 for all analyses).

**Table 5:** Unadjusted relationship between the public’s trust in various actors and reluctance to purchase (yes versus no, where yes is the event category)

Less likely to buy	Trust level	Health department			Scientists			Journalists			Politicians		
		Unadjusted odds ratio	99 % LCI	99 % UCI	Unadjusted odds ratio	99 % LCI	99 % UCI	Unadjusted odds ratio	99 % LCI	99 % UCI	Unadjusted odds ratio	99 % LCI	99 % UCI
Food	Low	1 (Ref)			1 (Ref)^			1 (Ref)			1 (Ref)		
	Moderate	0.75	0.45	1.24	0.75	0.42	1.36	1.03	0.67	1.59	1.01	0.62	1.66
	High	0.58	0.33	1.03	0.50	0.27	0.91	0.73	0.31	1.74	0.73	0.21	2.60
Sunscreens	Low	1 (Ref)^			1 (Ref)^			1 (Ref)			1 (Ref)		
	Moderate	0.56	0.36	0.82	0.55	0.33	0.91	0.90	0.61	1.32	0.89	0.57	1.40
	High	0.35	0.20	0.60	0.38	0.22	0.66	0.55	0.25	1.19	0.24	0.07	0.85
Medicines	Low	1 (Ref)^			1 (Ref)^			1 (Ref)			1 (Ref)^		
	Moderate	0.66	0.42	1.03	0.67	0.41	1.10	0.97	0.65	1.43	0.91	0.57	1.43
	High	0.53	0.31	0.90	0.44	0.26	0.75	0.50	0.23	1.09	0.23	0.07	0.75
Pesticides	Low	1 (Ref)			1 (Ref)^			1 (Ref)			1 (Ref)		
	Moderate	0.70	0.45	1.08	0.74	0.46	1.20	1.14	0.78	1.68	0.93	0.59	1.46
	High	0.57	0.34	0.97	0.53	0.32	0.88	1.00	0.45	2.22	0.31	0.08	1.20
Tennis racquets/computers	Low	1 (Ref)^			1 (Ref)^			1 (Ref)			1 (Ref)		
	Moderate	0.61	0.39	0.95	0.64	0.39	1.04	1.07	0.70	1.62	0.83	0.51	1.37
	High	0.40	0.22	0.70	0.40	0.22	0.69	1.46	0.65	3.28	0.20	0.04	1.14

^ Indicates that the overall trust factor is significant (*P* ≤ 0.01)

### Relationship between familiarity and reluctance to purchase

The unadjusted logistic analysis in Table 6 shows that when compared to other sections of the public, the section of the public that reported no familiarity with nanotechnology were less likely to buy off-the-shelf medicines and tennis racquets/computers if they were labelled as containing nanomaterials. However, when adjusting for risk perception, age and gender, we found no association between familiarity of nanotechnology and being less likely to buy a product if it had a label on it, while risk perception was significant for all analyses (*P* < 0.01).

**Table 6:** Relationship between familiarity and reluctance to purchase adjusted by age, gender and risk perception for public opinion only (yes versus no, where yes is the event category)

Label	Familiarity	Crude odds ratio	99 % LCI	99 % UCI	Adjusted odds ratio	99 % LCI	99 % UCI
Food Ref: ‘Yes’	No familiarity	1 (Ref)			1 (Ref)		
	Some familiarity	0.67	0.40	1.13	0.91	0.47	1.76
	Moderate familiarity	0.71	0.41	1.21	1.48	0.73	2.99
Sunscreen Ref: ‘Yes’	No familiarity	1 (Ref)			1 (Ref)		
	Some familiarity	0.79	0.50	1.24	1.28	0.70	2.36
	Moderate familiarity	0.67	0.42	1.08	1.27	0.69	2.35
Off the shelf medicines Ref: ‘Yes’	No familiarity	1 (Ref)^			1 (Ref)		
	Some familiarity	0.58	0.36	0.91	0.86	0.49	1.51
	Moderate familiarity	0.61	0.38	0.99	1.18	0.64	2.16
Pesticides Ref: ‘Yes’	No familiarity	1 (Ref)			1 (Ref)		
	Some familiarity	0.65	0.41	1.02	0.98	0.54	1.78
	Moderate familiarity	0.67	0.42	1.07	1.12	0.59	2.11
Tennis racquets/computers Ref: ‘Yes’	No familiarity	1 (Ref)^			1 (Ref)		
	Some familiarity	0.49	0.30	0.79	0.60	0.31	1.18
	Moderate familiarity	0.35	0.21	0.58	0.54	0.25	1.16

^ Indicates that the overall familiarity factor is significant (*P* ≤ 0.01)

## Discussion

The examination of stakeholder opinion is key to good policy as it ensures a holistic consideration of issues leading to a greater chance of policy acceptance. Within stakeholder opinion, the considerations of public opinion for nanotechnology policy development has been argued from a normative and substantive perspectives (Katz et al. 2009; Rogers-Hayden and Pidgeon 2007). Engaging the public contributes to greater transparency and enhanced democracy. It is one part of the politics of addressing controversial questions in a democratic society. All decisions have a social component, so by considering public opinion, a more holistic solution is found. In the area of nanotechnologies where there are many uncertainties regarding its risks, the public have a legitimate voice.

This study provides evidence to be used in the development of nanotechnology policy. It examines opinions regarding the use of labelling as a risk/policy tool for nanotechnologies in Australian society and explores the factors associated with these opinions. It investigates the differing risk and labelling perceptions between the public and academic, government and business figures involved in nanotechnologies in Australia.

This study found broad-based support for labelling of products containing nanomaterials. This is consistent with other studies (Brown and Kuzma 2013; IPSOS Social Research Institute 2012; Throne-Holst and Rip 2011) and applied across all stakeholder groups (public, academia, government, business). There was also evidence that labelling of products containing nanomaterials is likely to greatly influence consumer-purchasing behaviour. Food products are the items most sensitive to labelling, although off-the-shelf medicines, cosmetics/sunscreens and pesticides are likely to be affected by labelling. Consumer products such as tennis racquets and computers may be affected but not to the extent of the other products.

Our study found the public were less likely to buy a product labelled as containing nanomaterials than any other stakeholder, with the exception of tennis racquets/computers. This difference remained for public versus academic opinion for most products when we adjusted for risk perception. Thus, we were able to determine that risk perception alone was not the whole driver for the dissociation between public and academic attitudes around reluctance to purchase. Other factors including an involvement in nanotechnology play a role in shaping the acceptance of nano-labelled products. Given the small business and government sample sizes, it is not surprising that we could not detect a difference in reluctance to purchase between these stakeholders and the public after adjusting for risk perception. These underpowered samples made it unlikely to detect any true difference, if it existed.

For those members of the public that perceived nanomaterials as a risk in a product, labelling of that product indicating nanomaterials was associated with a reluctance to purchase. Consumers reported they were up to 24 times less likely to purchase depending on the product. However, there was a significant difference in the prevalence of those who were reluctant to purchase a labelled product when compared to the prevalence of those who thought the same product was a risk if it contained manufactured nanomaterials (Table 3). This suggests that a proportion of the public who thought the product was a risk would not be reluctant to purchase the product if it had a label on it stating it contained nanomaterials.

Our study found reduced trust in scientists and the health department was associated with a reluctance to purchase most products. However, after adjusting for age, gender and perception of risk, trust in any actor was no longer significant with perception of risk significant for all associations.

Increased trust in scientists, the health department and politicians has been associated with reduced perceptions of risk of manufactured nanomaterials (Capon et al. 2015a). Generically, work has been undertaken to determine the directions of the association between trust and risk perception. One direction is, does trust effect your perception of risk that in turn affects your purchasing behaviour (the casual ‘indirect’ model). The other direction is, does your perception of risk affect your trust in a regulator, and therefore, there is no relationship between trust and purchasing behaviour. Evidence exists for both models (Bronfman and Vázquez 2011; Eiser et al. 2002; Poortinga and Pidgeon 2005). Thus, we conclude that trust in scientists and the health department are not likely to have a direct influence over purchasing behaviour, rather may influence the risk perceptions of the public, which will then influence their purchasing behaviour. Therefore, trusted scientists and health department officials should communicate the risks and benefits of nanomaterials as a central part of any labelling strategy. In this way, interpretation of labelling issues that the public might voice may be indirectly addressed, allowing appropriate implementation.

The relationship between public familiarity with nanotechnology and reluctance to purchase appears similar to that of trust. Lower familiarity was associated with reluctance to purchase off-the-shelf medicines and tennis racquets/computers. However, after adjusting for age, gender and perception of risk, familiarity was no longer significant for any products, with perception of risk significant for all associations.

Increased familiarity with nanotechnology has been shown to be significantly associated with a reduced perception of risk of manufactured nanomaterials (Capon et al. 2015a). If we assume, like trust, that the direction of association between familiarity and risk perception is from familiarity to risk perception (causal chain), i.e. your familiarity with nanotechnology affects your risk perception then, like trust, familiarity would have an indirect effect on purchasing behaviour via risk perception. Therefore, in theory, increasing familiarity with nanotechnology could be one way of reducing possible public stigmatisation of manufactured nanomaterials from a labelling strategy. There are, however, limitations with the familiarity concept which have been highlighted elsewhere (Capon et al. 2015a).

This study extracted a number of issues with labelling, the most prominent regarding community perception. While some believed labelling would alarm the community and create a backlash against nanotechnology, others expressed labelling would create trust and social acceptance of nanotechnology. This study was not designed to determine which viewpoint would prevail. That would depend on the labelling and communication strategy that followed it. Further research into specific labelling messaging and positioning would need to be undertaken, and it is recommended that a labelling scheme, if it should be adopted, should have this research undertaken before implementation to ensure the labelling met its intended purpose.

A number of issues raised regarding the feasibility of a labelling scheme are the same issues that plague risk regulators in determining the safety of nanomaterials. Currently, there is a lack of appropriate or standard tests to identify or characterise many nanomaterials as well as a lack of toxicological data (Editorial 2011) and population exposure measurements (Beaudrie and Kandlikar 2011; Canady 2010; Maynard et al. 2011; Morris et al. 2011; Williams et al. 2010). Therefore, some of the problems surrounding the implementation of a labelling system are beyond the labelling scheme itself, rather inherent in introducing a new technology into society. With regard to population exposure measures, labelling, as was raised, is one solution in identifying who in the community is being exposed to certain manufactured nanomaterials, along with product registers.

This study has a number of strengths and limitations which have been covered in previous publications (Capon et al. 2015a, b) including the small number of respondents for business and government that provided little statistical power to determine associations. The response rate for these groups was similar or higher than for other groups, as the sample size was a result of the small number of people employed in these categories in Australia.

This study has a number of further limitations. Firstly, the study examines a consumer’s intention to buy and not the actual purchasing behaviour. Studies are inconclusive as to the relationship between intent and actual purchasing behaviour (Barber and Taylor 2013), and in reality, a number of factors such as price and choice will affect a consumer’s actual purchasing behaviour. Secondly, the study is limited by an inability to survey an actual labelling concept. The purchasing behaviour of consumers is likely to be influenced by the construction and positioning of the label itself. Positioning on front of label in a prominent location as was undertaken in (Siegrist and Keller 2011) has been shown to increase risk perception and likely affect consumer purchasing. Labelling placed at the back of the product, in the ingredient section, with the word (nano) next to the ingredient as undertaken in the EU has led to little societal response (Frewer et al. 2014), and it appears to have allowed consumers to become aware of nanoproducts without creating undue consumer concern.

## Conclusion

How the Australian public perceive the risk of nanomaterials within a particular product is the main driver in altering the public’s purchasing habits if that product is labelled as containing manufactured nanomaterials. The public are significantly more likely to want labelling of products that contain manufactured nanomaterials than those involved in nanotechnologies from academic, business and government sectors; however, overall support for labelling of products containing manufactured nanomaterials was high, irrespective of stakeholder. While risk perception was an important predictor of reluctance to purchase, it could not explain the entire difference between public and academic reluctance suggesting other factors are also driving the dissociation between stakeholder views on labelling. Food products are most likely to be affected by labelling, while consumer goods such as sporting equipment and computers are less so. Trust and familiarity were not found to have a direct effect on reluctance to purchase, but are likely to have an indirect effect through their association with risk perception. Consumer perceptions were the most mentioned reason for implementing and not implementing a labelling scheme. Therefore, understanding the public’s tolerance of risk for nanotechnologies and the factors that drive this risk will lead to greater success in implementing a labelling policy.
